# Metabolic and Transcriptomic Approaches of Chitosan and Water Stress on Polyphenolic and Terpenoid Components and Gene Expression in *Salvia abrotanoides* (Karl.) and *S. yangii*

**DOI:** 10.3390/ijms242015426

**Published:** 2023-10-21

**Authors:** Farzaneh Khodadadi, Farajollah Shahriai Ahmadi, Majid Talebi, Adam Matkowski, Antoni Szumny, Mahvash Afshari, Mehdi Rahimmalek

**Affiliations:** 1 Department of Plant Biotechnology, Ferdowsi University of Mashhad, Mashhad 91779-48974, Iran; khodadadi_far@yahoo.com; 2 Department of Plant Biotechnology and Plant Breeding, Ferdowsi University of Mashhad, Mashhad 91779-48974, Iran; shahriari@um.ac.ir; 3 Department of Biotechnology, College of Agriculture, Isfahan University of Technology, Isfahan 84156-83111, Iran; mtalebi@iut.ac.ir; 4 Department of Pharmaceutical Biology and Botany, Wroclaw Medical University, Borowska 211, 50-556 Wroclaw, Poland; pharmaceutical.biology@wp.eu; 5 Department of Food Chemistry and Biocatalysis, Wrocław University of Environmental and Life Sciences, 50-375 Wroclaw, Poland; 6 Department of Agronomy and Plant Breeding, College of Agriculture, Isfahan University of Technology, Isfahan 83111-84156, Iran; m.afshari1992@gmail.com; 7 Department of Horticulture, College of Agriculture, Isfahan University of Technology, Isfahan 84156-83111, Iran

**Keywords:** chitosan, drought stress, gene expression, HPLC, *Salvia*, tanshinone

## Abstract

In this research, a HPLC analysis, along with transcriptomics tools, was applied to evaluate chitosan and water stress for the prediction of phenolic flavonoids patterns and terpenoid components accumulation in *Salvia abrotanoides* Karel and *S. yangii*. The results indicated that the tanshinone contents under drought stress conditions increased 4.2-fold with increasing drought stress intensity in both species. The rosmarinic acid content in the leaves varied from 0.038 to 11.43 mg/g DW. In addition, the flavonoid content was increased (1.8 and 1.4-fold) under mild water deficit conditions with a moderate concentration of chitosan (100 mg L^−1^). The application of foliar chitosan at 100 and 200 mg L^−1^ under well-watered and mild stress conditions led to increases in hydroxyl cryptotanshinone (OH-CT) and cryptotanshinone (CT) contents as the major terpenoid components in both species. The expressions of the studied genes (DXS2, HMGR, KSL, 4CL, and TAT) were also noticeably induced by water deficit and variably modulated by the treatment with chitosan. According to our findings, both the drought stress and the application of foliar chitosan altered the expression levels of certain genes. Specifically, we observed changes in the expression levels of DXS and HMGR, which are upstream genes in the MEP and MVA pathways, respectively. Additionally, the expression level of KSL, a downstream gene involved in diterpenoid synthesis, was also affected. Finally, the present investigation confirmed that chitosan treatments and water stress were affected in both the methylerythritol phosphate pathway (MEP) and mevalonate (MVA) pathways, but their commitment to the production of other isoprenoids has to be considered and discussed.

## 1. Introduction

*Salvia* is one of the largest and most important genera of medicinal and aromatic plants that has been both cultivated on an industrial scale and collected from natural habitats. West and central Asia are among the biodiversity hotspots of *Salvia*, with a significant importance both for local folk medicine as well as the food industries. The *Perovskia* of the extended genus of *Salvia* comprises several medicinal and aromatic species, of which *S. yangii* BT. Drew (formerly *Perovskia atriplicifolia* Benth.) and *S. abrotanoides* (Kar.) Systma (previously known as *Perovskia abrotanoides* Kar.) are mostly distributed in Iran, Turkmenistan, Pakistan, and Afghanistan [1]. Several applications have been reported for the treatment of common diseases such as leishmaniasis [2], neurodegenerative diseases such as Alzheimer’s disease [3], and liver fibrosis [4], as well as for rheumatic pain, headache, atherosclerosis, cough, and as an anti-diabetic [5,6].

The aerial parts of the *Salvia* genus have considerable polyphenols and some of them are critical in the food industries [7]. Moreover, the roots of several *Salvia* species, including members of the *Perovskia* subgenus, contain a class of a diterpene component called tanshinone, including tanshinone I (T-I), tanshinone IIA (T-IIA), dihydro-tanshinone I (DT-I), hydroxy-cryptotanshinone (OH-CT), cryptotanshinone (CT), and dihydrotanshinone (DT) as the major bioactive components [8,9]. Earlier studies have reported that the above-mentioned compounds have exhibited some biological activities, including antibacterial, anticancer, effects on heart function, and antioxidant properties [10].

Plants experience different environmental stresses, with drought being a particularly strong stress that greatly impacts their productivity. As water makes up a large proportion of a plant’s biomass, it plays a vital role in various physiological processes and in growth, development, and metabolism. Drought is recognized as a primary environmental stress, especially in dry regions, and is regarded as a significant threat to global food security [11]. The occurrence of oxidative stress is a notable consequence of drought stress [12], wherein the intensity of the damage inflicted by reactive oxygen species (ROS) as the primary physiological response is reliant on the efficiency of antioxidant defense systems. Alongside prevalent compounds such as ascorbic acid and glutathione, low-molecular-weight antioxidants encompass specialized metabolites such as polyphenols [13]. These compounds can prevent ROS damage in plants, and also have the capacity to mitigate many oxidative-stress-associated complaints in humans [14]. The physiological role of tanshinones in plants has remained largely unresolved to date, despite a huge body of evidence of their pharmacological activities in animal organisms.

Bio-elicitors are applied to soil or plants to enhance their physiological stress resilience [15]. Chitosan, derived from chitins, is a natural biopolymer and bio-elicitor that exhibits promising potential as a biostimulant and elicitor in agriculture. Notably, it possesses non-toxic, biodegradable, and biocompatible properties, making it highly suitable for a wide range of applications. By activating the stress transduction pathway through secondary messenger(s), chitosan enhances physiological responses and alleviates the negative impact of abiotic stresses [12,16]. Furthermore, chitosan was selected as an elicitor to enhance the production of phenolics and diterpenoids in plants under water deficit [17]. The effects of chitosan on drought stress have been used in various plants such as maize [18], *Artemisia annua* [19], *Sorghum* [20], and *Salvia* [21,22], and also, in most cases, it reduces the effects of drought stress.

The biosynthetic pathways of phenolic acids are well elucidated, as they are produced via the phenylpropanoid pathway. Rate-limiting enzymes, Phenylalanine ammonia-lyase (PAL), tyrosine aminotransferase (TAT), cinnamic acid 4-hydroxylase (C4H), hydroxyphenylpyruvate reductase (HPPR), rosmarinic acid synthase (RAS), coenzyme A ligase (4CL), and *CYP98A14* have been identified in phenolic compounds. Diterpenoids’ basic building blocks—active isoprene units—are formed via the cytosolic mevalonate (MVA) pathway and the plastidic methylerythritol phosphate (MEP) pathway in plastids [23]. The key enzyme genes of the general isoprenoid pathway include in the MVA branch, 3-hydroxy-3-methylglutaryl CoA reductase (HMGR), and in the MEP branch, 1-deoxy-D-xylulose 5-phosphate synthase (DXS) and 1-deoxy-D-xylulose 5-phosphate reductoisomerase (DXR). The diterpene scaffold and its further modifications are assembled by geranylgeranyl diphosphate synthase (GGPPS), copalyl diphosphate synthase (CPS), kaurene synthase-like (KSL), and a cytochrome P450 enzyme (*CYP76AH1*) that have been characterized through cloning and transcriptomic approaches [24,25]. Besides some studies on *Salvia milirtioza* tanchinone compounds [23,25], there is no comprehensive research regarding tanchinone alternations in respect to molecular and metabolites points of view in *S. abratonoides* and *S. yangii*.

However, the downstream tanshinone-specific pathway also remains to be described. Therefore, the main aim of the present study was to determine the effects of drought stress and chitosan treatment on the polyphenol and thanshinone contents in *S. abrotanoides* and *S. yangii*. Additionally, the expressions of several genes related to the biosynthesis of the tanshinone and phenolic acids in the studied species under drought stress and foliar treatments were investigated.

## 2. Results and Discussion

### 2.1. Results

#### 2.1.1. Variance Analysis of Phytochemical Traits

The results of the variance analysis are presented in Table 1. The results showed that drought stress had a significant effect on the total tanshinone content (TTC), total phenolic root content (TPC_root_), and total flavonoid leaf content (TFC_leaf_) (Table 1). In addition, the chitosan application had a significant effect on all the studied traits except the TPC in the root. Additionally, the drought stress and chitosan interaction showed a significant effect on all traits. Accordingly, the effect of species in terms of the chitosan interaction was not significant for TFC_root_, TFC_leaf_, and TPC_leaf_. Finally, there was no significant interaction between species× stress× chitosan for TTC and the total flavonoids of the roots (Table 1).

#### 2.1.2. Total Phenolic Content of Roots and Leaves

Based on the variance analysis, the leaf TPCs were significantly different for the chitosan spray and chitosan× stress interaction. On the contrary, the effect of the stress levels and drought × chitosan foliar interaction were significant in the root TPC (Table 1). The total phenolics were significantly higher in the leaves than in the roots. The highest (57.18 mg TAE/g DW) amount of TPC in the leaves was observed in the non-stressed plants with 100 mg L^−1^ of chitosan foliar application (Table 2A). Furthermore, upon the application of 200 mg L^−1^ of foliar chitosan under severe drought stress, the phenolic content increased from 36.85 to 56.36 mg of TAE/g of DW. A non-significant decrease in TPCleaf was observed with an increasing chitosan concentration in the mildly stressed plants. The TPCs in the leaf were recorded at 49.05, 45.89, and 44.26 mg of TAE/g DW at a mild stress level when treated with 0, 100, and 200 mg of L^−1^ chitosan, respectively. Hence, under severe drought stress, the phenolic compounds acclimation decreased with the application of 200 mg L^−1^ of chitosan (Table 2A). *S. yangii* had the highest (53.05 mg TAE/g DW) value of the TPCleaf extract under the control conditions (Table 2B). In relation to the TPC of the root extract, the data analysis showed that the highest (15.38 mg of TAE/g of DW) and lowest (8.65 mg of TAE/g of DW) values under drought stress conditions were obtained with the application of 100 mg L^−1^ of foliar chitosan (Table 2A).

#### 2.1.3. Total Flavonoid Content

Under drought stress, there was no significant difference in the TFC in the leaves, but the chitosan application and interaction of chitosan spray× stress were significantly different. Furthermore, significant differences in the TFC in the root samples were shown under drought stress, chitosan spray, and two-way interaction (Table 1). The highest accumulation of TFCleaf (5.04 mg QE/g DW) was obtained in the no stress condition using 100 mg L^−1^ of foliar chitosan application (Table 2A). The 100 mg L^−1^ chitosan foliar applications displayed an increase in the TFC under both drought stress and well-watered conditions compared to the lack of chitosan (Table 2A). Under non-stressed conditions, the flavonoid content increased with a high concentration of chitosan relative to the control, where no chitosan was applied. Interestingly, the chitosan treatments under severe drought stress resulted in a reduction in TFC in the root samples (Table 2A). *S. abrotanoides* had the highest amount of TFCroot under non-stressed conditions (Table 2B).

#### 2.1.4. Total Tanshinone Content (TTC) and HPLC Analysis

An analysis of variance showed that the TTC significantly differed in terms of the foliar chitosan application, drought stress, and chitosan × drought interaction effects (Table 1). As seen in Table 2A, the content of total tanshinones ranged from 11.16 to 14.01 mg/g DW in *S. abrotanoides* and from 9.93 to 21.23 mg/g DW in *S. yangii* according to the spectrophotometric method. The TTC increased under well-watered conditions and moderate stress with the application of chitosan (200 mg L^−1^). The usage of chitosan in the treated plants under severe drought stress conditions led to a decrease in the TTC value. When using 0 mg L^−1^ of chitosan, the tanshinone content increased in the average of two species under severe drought stress (15.47 mg/g of DW) (Table 2A).

Substantial variation among the phenolic acid and tanshinone components was observed under the control and drought stress conditions with the chitosan treatments. The results of the HPLC analysis are presented in Table 3. Five phenolic compounds: apigenin, caffeic acid, chlorogenic acid gallic acid, and rosmarinic acid, and three tanshinones: OH-CT, CT, and T-IIA, were identified using HPLC in the roots and leaves. In the present study, different responses were observed for *S. abrotanoides* and *S. yangii* in terms of the chitosan treatments and stress and non-stress conditions. As seen in Table 3, the T-IIA content ranged from 0.45 to 1.75 mg/g DW in *S.yangii* and from 0.27 to 1.12 mg/g DW in *S. abrotanoides*. The highest amount of T-IIA was recorded in *S. yangii* under severe stress with 0 mg of L^−1^ foliar chitosan.

The application of foliar chitosan at 100 and 200 mg L^−1^ under well-watered and mild stress conditions led to increases in the OH-CT and CT contents in both species. Interestingly, a different response was observed in the two species studied under severe stress in relation to the foliar application. Under severe drought stress, *S. yangii* showed high levels of OH-CT and CT with an increasing chitosan concentration, whereas the trend for *S. abrotanoides* was quite inversed (Table 3). Under severe drought stress, the main compounds of *S. abrotanoides*, such as CT (1.277 mg/g DW), were increased without foliar chitosan usage, whereas this component decreased under the chitosan treatments (100 and 200 mg L^−1^).

The rosmarinic acid content in the leaves ranged from 0.038 mg/g DW in *S. yangii* under 100 mg L^−1^ of foliar chitosan application and severe stress to 11.43 mg/g DW in *S. abrotanoides* under control conditions with 100 mg L^−1^ of chitosan foliar application (Table 3). The same trend was observed in the roots.

#### 2.1.5. Principle Component Analysis (PCA)

**Figure 1:**
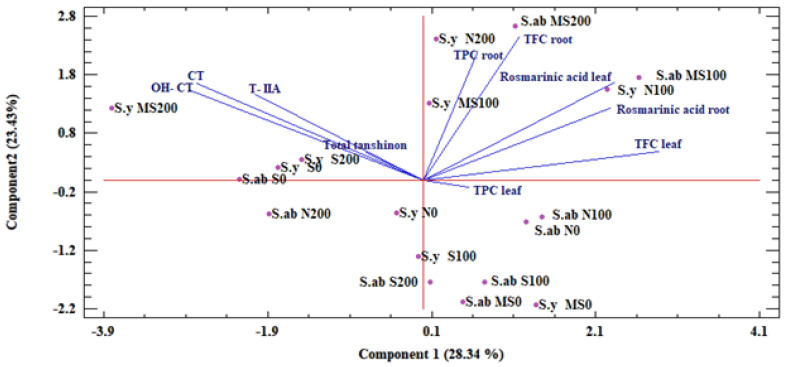
The principal component analysis (PCA) based on total phenolic, total flavonoid, and total tanshinone contents, individual tanshinone and rosmarinic acid under drought stress conditions in both Salvia species (S.y: *S. yangii*; S.ab: *S. abrotanoides*) with different concentrations of chitosan. N: well-watered condition; MS: mild stress condition; S: severe stress condition; 0, 100, and 200 mg concentrations of chitosan L^−1^. TPC: total phenolic content; TFC: total flavonoid content; T−IIA: Tanshinone−IIA; CT: Cryptotanshinone; and OH−CT: Hydroxy−cryptotanshinone.

Accordingly, PC_1_ explained 28.34% of the total variance in the dataset, whereas the second PC (PC_2_) showed 23.43% of the variance (Figure 1). The PC_1_ had a positive correlation for the rosmarinic acid in leaves, rosmarinic acid in roots, TPCleaf, TPCroot, TFCleaf, and TFCroot, as well as negative loading for the TTC, T-IIA, OH-CT, and CT components. The PC_2_ was positively correlated with all the mentioned traits except TPCleaf. 

#### 2.1.6. Gene Expression

**Figure 2:**
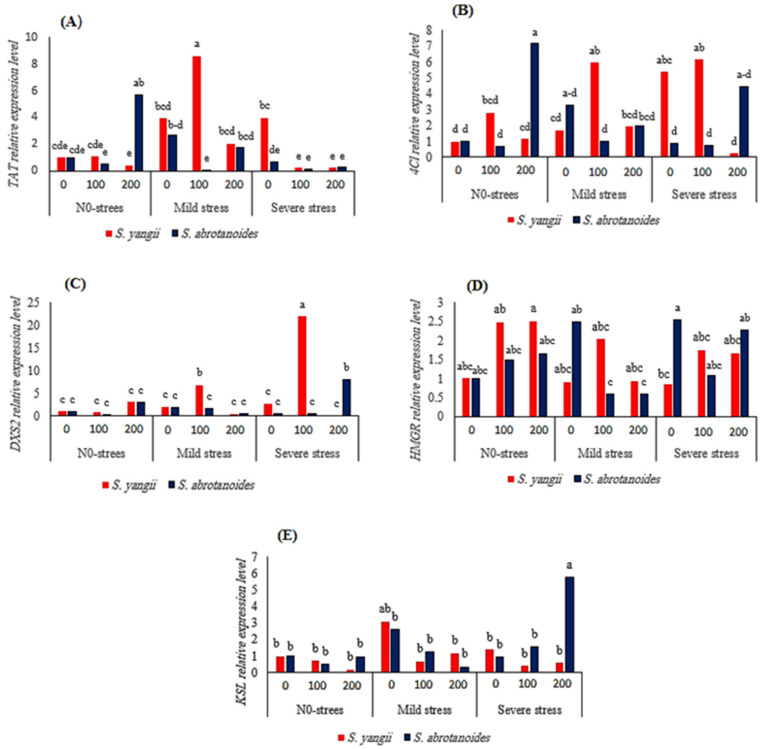
(**A**–**E**) Relative gene expression analysis in root tissue of *Salvia abrotanoides* and *S. yangii*, subjected to well-watered and drought conditions at different chitosan concentrations. 4CL: 4-Coumarate-CoA ligase gene; TAT: Tyrosine aminotransferase gene; KSL: Kaurene synthase-like; HMGR: 3-Hydroxy-3-methylglutaryl CoA reductase; and DXS: 1-Deoxy-D-xylulose 5-phosphate synthase. In each column, the means followed by the same letter are not significantly different according to the LSD test at 0.05.

The 4CL and TAT genes’ expression levels were determined because of their involvement in the phenolic acid biosynthetic pathways (Figure 2). The expression levels of all the studied genes were noticeably induced by the chitosan foliar application and drought stress. Regarding the phenylpropanoid pathway, the 4CL expression levels of 4CL reached their maxima (7.14-fold) with the foliar application of chitosan (200 mg L^−1^) under well-watered conditions in *S. abrotanoides*, whereas the TAT expression increased 8.62-fold with the chitosan treatment (100 mg L^−1^) under moderate drought stress in comparison to the control samples. Furthermore, these changes in the TAT expression under drought stress were much higher in *S. yangii* than in *S. abrotanoides* (Figure 2). Shabani et al. showed that the TAT expression in *Melissa officinalis* was enhanced at all concentrations (50, 100, and 150 mg L^−1^) of chitosan compared to that under control conditions [26].

**Figure 3:**
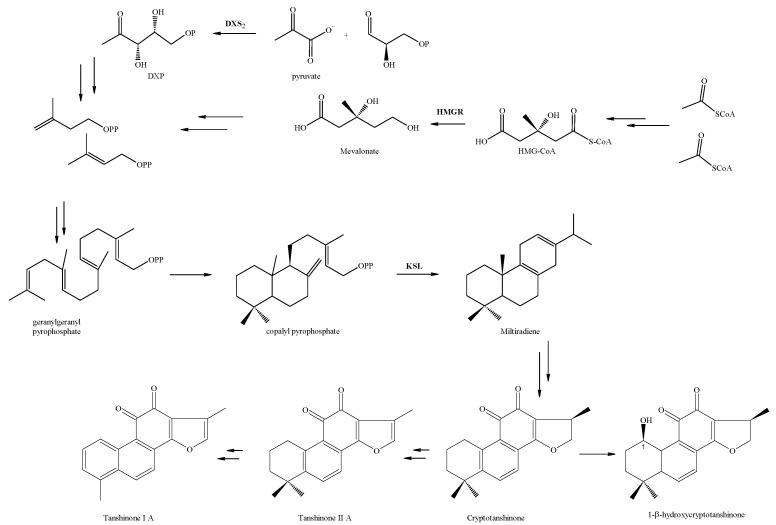
Biosynthetic pathway of tannshinones. KSL—kaurene synthase-like; DXS2—1-Deoxy-D-Xylulose 5−Phosphate Synthase 2; and HMGR−3−hydroxy-3−methyl−glutaryl−reductase.

**Figure 4:**
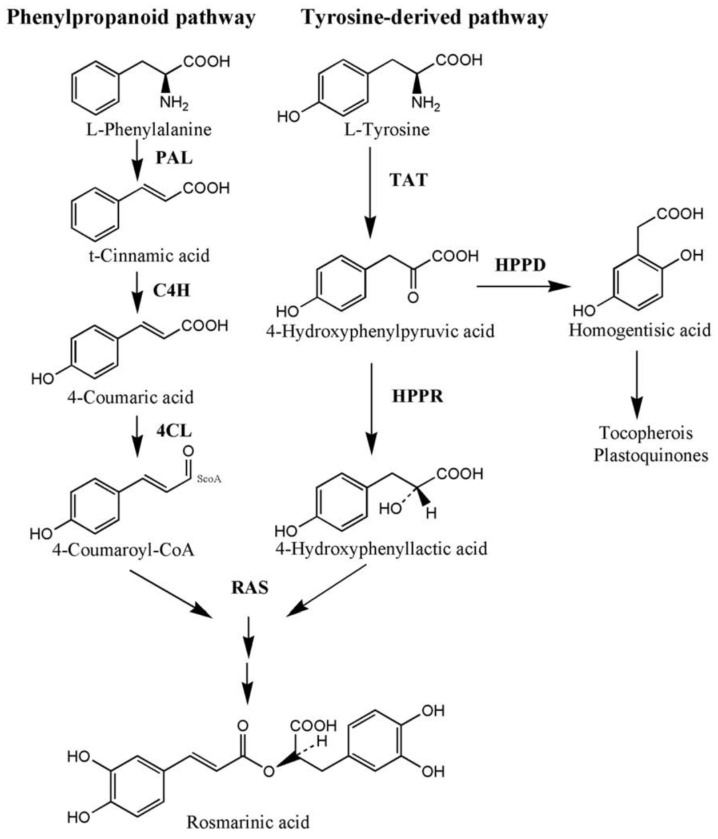
Biosynthetic pathway of rosmarinic acid. PAL; phenylalanine ammonia-lyase, C4H; cinnamic acid 4-hydroxylase, 4CL; hydroxycinnamate coenzyme A ligase, TAT; tyrosine aminotransferase, HPPR; 4-hydroxyphenylpyruvate reductase, RAS; RA synthase, and HPPD; 4-hydroxyphenylpyruvate dioxygenase [27].

Our results showed that the drought stress and foliar chitosan application changed the expression levels of the DXS, HMGR (as upstream genes of the MEP and MVA pathways, respectively), and KSL (as a downstream specific gene of diterpenoid) genes (Figure 1). The DXS2 activity of the severe water stressed groups tended to increase dramatically (Figure 3 and Figure 4) and reached a maximum at 100 mg L^−1^ of chitosan, which was 20.90-fold in *S. yangii*, whereas *KSL* and *HMGR* reached their maximums in *S. abrotanoides* at a level of severe water stress with applications of 200 and 0 mg L^−1^ of chitosan. The transcript level of *HMGR* increased significantly by 2.51-fold in *S. yangii* and 1.64-fold in *S. abrotanoides* at a high level of chitosan under well-watered conditions compared to the control samples (Figure 2).

#### 2.1.7. Correlation Analysis

Simple correlation coefficients were calculated among the noted characteristics to determine their relationships with each other (Table 4). Higher correlation coefficients in the correlation matrix were noticed between OH-CT and CT (0.86 **), followed by T-IIA and CT (0.54 *), and T-IIA and OH-CT (0.48 *). The correlation of 4CL with the transcription of the DXS2 (0.62 **) and TAT (0.62 **) genes was positive (Table 4). Furthermore, there was a weak negative correlation between TPCroot and TPCleaf (−0.18). The decreased TPC in the roots may have been due to the higher accumulation of TPC in the leaves, as they maintained a negative correlation with each other (Table 2B).

### 2.2. Discussion

#### 2.2.1. Total Phenolic Compounds and Total Flavonoid Contents

Reducing a plant’s access to water resources and being affected by bio-elicitors, including chitosan, can trigger alterations in many morphological, biochemical, and molecular physiological aspects in plants. These changes can have significant effects on various aspects of plant growth and development [27]. The results of the variance analysis demonstrated that both the drought stress and chitosan application had significant effects on the studied traits, such TTC, TPC, and TFC, with some variations depending on the specific trait and plant species. Drought stress influenced the TTC, TPCroot, and TFCleaf, while the chitosan application affected all the traits except TPCroot.

The variance analysis showed the significant effects of the chitosan spray and chitosan × stress interaction on the TPCleaf. In the *S. abrotanoides* extract, the highest phenolic content in the roots was detected under all the conditions studied (Table 2B,C), which could hypothetically have been due to the higher genetic potential of *S. abrotanoides* in TPC biosynthesis and the accumulation and lower effectiveness of environmental factors such as drought stress and the application of chitosan. Ali et al. proposed that a plant’s capacity for drought stress and metabolite changes in response to environmental stresses depends on the plant species [28].

One of the important and well-known functions of phenols is to participate in non-enzymatic defense mechanisms to deal with oxidative stress induced under drought stress conditions. These compounds act as free radical scavengers and increase a plant’s tolerance to oxidative stress [29]. In general, some herbs under drought conditions produce more phenol groups or single compounds to protect cells from oxidative injury [30]. For example, in peppermint, the foliar application of chitosan caused increases in antioxidant activity and phenolic content [31]. Vosoughi, Gomarian, Pirbalouti, Khaghani, and Malekpoor (2018) observed that the amounts of total phenolic and flavonoid content in sage were enhanced under water-limited conditions, when the plants were sprayed with chitosan [26]. Bistgani et al. reported that chitosan treatment (400 μL L^−1^) improved the biosynthesis of the phenols in *Thymus daenensis* [17]. In the same study, mild drought stress caused an increase in the phenolic compound content in *T. daenensis*, which agrees with our results. In contrast to our study, an increase in TPC as a response to drought stress did not occur in *Lactuca sativa* L. [30]. The earlier studies on Melissa. officinalis revealed that the highest phenolic compound content was found in the plants treated with 100 mg L^−1^ of chitosan [32]. However, the response of TPC to drought stress can vary among different plant species. The current result also showed that the highest value of TPC was recorded in the 100 mg L^−1^ chitosan-treated, well-watered plants (Table 2B). Overall, these findings contribute to our understanding of the effects of chitosan and drought stress on the phenolic compound accumulation in plants.

According to the previous report, the foliar application of chitosan as an elicitor induces the enzymes phenylalanine ammonia lyase and chalcone synthase, which, in turn, increase the contents of phenolics and flavonoids in *Artemisia aucheri* [33]. Furthermore, Amkha et al. reported that chitosan application at a moderate (120 mg L^−1^) versus high concentration (240 mg L^−1^) enhanced the total flavonoid and phenolic contents 1.11- and 2.37–4.90-fold, respectively [34]. In contrast to the present study, Afshari et al. reported that the TFC was higher in most of the *S. yangii* samples than the *S. abrotanoides* samples under severe water stress conditions [10]. The different results from previous studies suggest species-specific differences in flavonoid contents. In line with our results is the study on *Thymus*, where well-watered or mildly drought stressed plants acclimated flavonoids at the highest level [17]. Chitosan, as a generally acknowledged stress modulator, may display diverse effects on the adaptation of plants to stress, depending on plant species, application method, concentration, and time of application [35]. Overall, these findings contribute to our understanding of the effects of chitosan and drought stress on the flavonoid accumulation in plants.

#### 2.2.2. HPLC

In wild-growing *S. abrotanoides*, 30.67 μg/g DW of T-IIA content was reported. Furthermore, the highest contents of OH-CT and CT were reported for *S. yangii* under well-watered and severe stress conditions with 200 mg of foliar chitosan [9].

Putatively, stimulating tanshinone accumulation under water deficit conditions can help to minimize the harmful effects of ROS [36]. De Abreu and Mazzafera showed that the growth of plants under drought stress can cause significant increases in some secondary metabolites in plants [37]. Liu et al. reported enhanced tanshinones accumulation in *S. miltiorrhiza* under water deficit, which corroborates with our results [34]. Additionally, an increase in tanshinone content in response to hyperosmotic stress in *S. miltiorrhiza* has been confirmed in previous studies [38]. The response of tanshinone content to chitosan and drought stress can vary among species and may be influenced by geographical factors. Chitosan, as a stress modulator, can have diverse effects on plant adaptation to stress depending on various factors.

Caffeic acid derivatives constitute most of the phenolic acids in the *Salvia* genus [39]. Kamatou et al. showed that *S. albicaulis*, *S. runcinate*, and *S. muirii* are rich in rosmarinic acid [40]. In the present study, a higher level of caffeic acid was detected in *S. yangii* under severe stress conditions. However, caffeic acid derivatives occur mainly in the dimer form as rosmarinic acid [40]. Accordingly, Janicsák et al. reported that the caffeic acid concentrations in *Salvia* species are always much lower than those of rosmarinic acid [41]. According to previous studies, the tanshinone content may be markedly different in the same species based on geographical region. On the other hand, interspecific variation most likely influences the metabolic response to environmental stress [42].

#### 2.2.3. Gene Expression

The present investigation confirmed that the water stress and chitosan foliar treatments activated important tanshinone biosynthesis pathways, including the MEP and MVA pathways, but their commitment to the production of other isoprenoids has to be considered, too. However, the MEP pathway is generally believed to be more important than the MVA pathway in tanshinone biosynthesis [25]. Overall, this study provides insights into the regulatory mechanisms of tanshinone biosynthesis in response to chitosan and drought stress. The positive correlations between 4CL and the transcription of the DXS2 and TAT genes indicate a potential regulatory relationship between these genes in the biosynthesis pathway. Additionally, a weak negative correlation was observed between the TPC in the root and the TPC in the leaf, suggesting a possible trade-off in the accumulation of the total phenolic compounds between these two plant parts. These findings provide insights into the interrelationships among the studied characteristics and contribute to our understanding of the biosynthetic pathways and distribution patterns of the phenolic compounds in the plant.

#### 2.2.4. PCA Analysis

A PCA was applied to demonstrate the contribution of the experimental treatments to the variation with respect to the phytochemical traits of the leaves and roots under stress and well-watered conditions, with different concentrations in both species. The obvious separation between the two species was identified by analyzing the relationships of the studied variables based on the first two PCs. The PCA biplot analysis showed that there were three main groups, which were mainly divided based on their main polyphenol components and biochemical traits. Accordingly, the first group was characterized by *S. yangii* and *S. abrotanoides* under well-watered and mild stress conditions with chitosan foliar applications. These samples showed high values of rosmarinic acid root, rosmarinic acid leaf, TFCroot, TFCleaf, and TPCroot. The samples of the second group contained high levels of TPCleaf under well-watered and both mild and severe stress conditions with foliar applications of 0 and 100 mg L^−1^ of chitosan. The samples of the third group were distinguished by tanshinone components and TTC under stress conditions (Figure 1).

## 3. Materials and Methods

### 3.1. Ethics Statement

This study was carried out in accordance with the Ethics Committee of Isfahan University of Technology, Iran. In this regard, written and informed consent was obtained from all volunteers. In addition, the Ethical Committee of Isfahan University of Technology approved all the experimental protocols by monitoring them.

### 3.2. Plant Materials

The seeds of two populations of *Salvia* were obtained through Isfahan University of Technology, Isfahan, Iran. These two species, *S. abrotanoides* and *S. yangii*, were collected from the Khorasan Razavi and Sistan and Baluchestan provinces, respectively, by Dr. Zahra Ghaffari. Flora Iranica was used by Dr. Mehdi Rahimmalek (Isfahan University of Technology, Isfahan, Iran) to identify the plant species [43]. The voucher specimens are preserved in the Herbarium of Isfahan University of Technology under numbers 13363 and 13368 for *S. abrotanoides* and *S. yangii*, respectively. For further studies, in March 2018, the seeds were planted in pots with heights of 25 × 25 cm and diameters of 25 cm. Each pot contained 10 kg of soil with a mixture of sand and loam in a volume ratio of 1:2. The plants were kept under greenhouse conditions in 15 h light and 9 h dark cycles under ambient temperature in Isfahan University of Technology, Iran, for two months. The pots were transferred to the environment outside the greenhouse until April of the following year. In the second year, the soil mixture was changed to 53% sand, 33% silt, and 14% loam. In this year, the plants were grown under well-watered conditions from April 5th to May 5th. Then, all the pots were transferred to the same greenhouse conditions as previously. After one month, the irrigation regime of the plants was started. At the same time, various amounts of chitosan solution were sprayed every five days. The plants were treated with water deficit and chitosan for two months, and then leaf and root samples were collected separately. The collected leaves and roots were frozen in liquid N2 after being rinsed with distilled water. The frozen samples were stored long-term at −80 °C. Furthermore, to prepare the methanolic extract, 5 to 10 grams of leaf and root tissue were collected and dried in a dark room with ambient temperature for one month.

This study was conducted in summer 2019 with 18–28 °C daily temperatures and a 14 h photoperiod in Chah anari greenhouse at Isfahan University of technology, Isfahan, Iran.

### 3.3. Experimental Design and Treatments

In the present study, an experimental design was planned as a factorial randomized complete design (RCD) with three replications. The studied plants were exposed to three drought stress levels: (i) non-stress level (control), (ii) mild stress level, and (iii) severe stress level along with three chitosan values (0, 100, and 200 mg L^−1^). Irrigation was conducted every other day, every three days, and every five days as a control (well-watered), under moderate and severe stress, respectively. The amount of water given to each pot was calculated according to the field capacity of the vase. For this purpose, soil samples were collected and weighed from a depth of 10 cm from the center of each pot on days 0, 3, 5, 7, and 9 after irrigation and were considered as fresh weight (FW). Dry weight (DW) was determined after 48 hours of oven drying the soils at a temperature of 105 °C. The total SWC was calculated based on Formula (1) [44], as follows:

SWC (%) = [(FW − DW)/DW] × 100 (1)

Citric acid was used as a solvent of chitosan; thus, the control pots were foliarly sprayed with citric acid. The *Salvia* plants were sampled at the flowering stage, which was two months after the treatment. Weed control was performed manually during the experiment. In addition, no pesticides or fertilizers were given to the studied plants.

### 3.4. Total Phenolic (TPC) and Flavonoid Contents (TFC) Assays

The amount of TPC was measured using the Folin–Ciocalteu reagent according to the method of Afshari et al., with minor modifications [10]. To prepare a methanolic extract of the *Salvia* species, the dried leaves and roots of sage were powdered and extracted three times with methanol. For each sample, 250 mg of dry plant powder was extracted with 10 mL of 80% aqueous methanol using a shaker (110 rpm at 25 °C for 24 h). The obtained solution was filtered and 2.5 mL of the Folin–Ciocalteu reagent (1:10 diluted with distilled water) and 2 mL of a 7.5% sodium carbonate anhydrous solution were added to 0.5 mL of the methanolic extract. Finally, the mixture was incubated at 45 °C for 15 min. The absorbance of the solution was read at 765 nm against a blank using a Hitachi f-2500 fluorescence spectrophotometer. The value of TPC was expressed in mg of tannic acid equivalent (TAE) per gram of each extract.

The aluminum chloride colorimetric procedure was used to investigate the TFC [45] with some changes. Briefly, 125 µL of the extract (1:10 diluted with distilled water) was mixed with 300 µL of a 5% NaNO_2_ solution and incubated for 5 min, followed by mixing with 600 µL of AlCl_3_ (10% *w*/*v* in distilled water). In the end, the samples were mixed with 2000 µL of NaOH (1 M), and 2000 µL of distilled water was used to reach the final volume of the solution. The absorbance was recorded at 510 nm against a blank. The total flavonoid content (TFC) was presented in mg of quercetin equivalents (QE) per gram of the extract on a dry basis. All the determinations of the TPC and TFC were carried out in triplicate. All the commercial salts and reagents were purchased from Merck and Sigma-Aldrich, St. Louis, MO, USA.

### 3.5. Total Tanshinone Content (TTC) and High-Performance Liquid Chromatography (HPLC) Analysis

The extraction was performed according to the method of Afshari et al. [10]. One gram of dry root samples of the *salvia* species was completely powdered using a mortar and pestle. A total of 10 mL of HPLC-gradient grade methanol was added to each root sample and incubated for 4 h in a water bath at 35 °C. Whatman filter papers (No.1) were used to filter the crude extracts three times. The absorbance of the samples was recorded at 270 nm as the total tanshinone content (TTC). The methanol extract of each sample was filtered and then evaporated under reduced pressure to dryness. For the HPLC analysis, the crude extracts were filtered through a 0.22 μm micropore membrane, and then 20 µL of each sample was injected into the Agilent model HPLC system (Agilent 1090 Series) at 25 °C. A HPLC symmetry C18 column (Waters, Milford, MA, USA, with a 250 × 4.6 mm, 5 µm particle size) was applied using a binary solvent system of 0.1% formic acid/water and 0.1% formic acid/acetonitrile as solvents A and B, respectively, at flow rate of 0.8 mL min^−1^. A five-step gradient elution protocol was employed as follows: at the beginning (in 0 min), 60:40 solvent A/B (*v*/*v*), at 5 min, 40:60 solvent A/B, at 20 min, 40:60 solvent A/B, at 23 min, 20:80 solvent A/B, and finally (at 25 min), 0:100 solvent A/B. Then, the HPLC chromatogram absorbance at 270 nm was obtained. All standards (gallic acid, rosmarinic acid, apigenin, caffeic acid, chlorogenic acid, OH-CT (CAS 18887-18-8), CT (CAS 35825-57-1), and T-IIA (CAS 568-72-9)) were dissolved in HPLC-grade methanol with a range of concentrations between 0.2 and 20 mg/L before being injected into the analytical HPLC system. The contents of tanshinones and polyphenols were expressed as mg/g DW (mg per g of sample dry weight) and validated by authentic standards [46].

### 3.6. Isolation of Total RNA, cDNA Synthesis, and Real-Time PCR

Roots of the *Salvia* species were collected and 100 mg of each sample was pulverized using a cold mortar and pestle to extract the total RNA using the cetyltrimethylammonium bromide (CTAB) method with minor modifications [46]. DNA contamination of all the samples was eliminated with DNase I (Promega, USA) for 30 min according to the manufacturer’s protocol. The RNA concentration and purity of each RNA sample were evaluated using Nanodrop (IMPLEN, NP80, Germany) at 260 nm. To obtain the cDNA, the total RNA (1 μg) was reverse transcribed to cDNA using a cDNA synthesis kit (Yekta Tajhiz Azma, St. Rashid, Tehran, Iran), according to the manufacturer’s protocol. The Step One Real-Time PCR System (Applied Biosystems, Waltham, Massachusets, USA) was used for the real-time PCR, with a reaction volume of 10 μL in each reaction mixture. The real-time PCR was programed for 40 cycles. Before starting the real-time cycles, the initial denaturation was performed for 10 min at 95 °C. The main cycles were followed by 95 °C for 15 s (denaturation), 60 °C for 25 s (primer annealing), and 95 °C for 5 s (primer extension). The comparative Ct method (2^−ΔΔCt^), considering the expression level of the elongation factor as a housekeeping gene, was used to investigate and analyze the relative transcript levels of the genes with three independent biological replicates and three technical replicates.

The expressions of the genes related to tanshinone (DXS2, HMGR, and KSL) and the phenolic acid biosynthetic pathway genes (4CL and TAT) were analyzed in the two species studied under different water regimes and concentrations of chitosan.The primers were designed, using the information available in the NCBI database for the DXS2, HMGR, and KSL genes in the Salvia family, to determine part of the sequence of these genes in Salvia. Then, based on the sequencing, real-time primers were designed using the Oligo 7 software. The primers related to the TAT and 4CL genes [47] and the Elf primer [10] related to the internal control were extracted from the previous study (Table 5).

### 3.7. Statistical Analysis

The data were analyzed based on factorial experiments in a completely randomized design using SAS version 9.4. (SAS Institute, Cary, NC, USA) with three replications. A means comparison was performed using the Duncan’s multiple range test at a confidence level of 95%. A Principal Component Analysis (PCA) was carried out to determine the associations between the variables using the Statgraphics software version 18.2.04.

## 4. Conclusions

In the two Iranian *Salvia* species, naturally adapted to extreme changes in water availability, the foliar application of chitosan significantly modulated the effect of water deficit stress. Under a specific combination of stress and elicitor treatment, enhanced contents of phenolic compounds and pharmacologically valuable tanshinones could be achieved. The total phenolic content of the leaves decreased with an increasing chitosan concentration under moderate drought stress. Additionally, under severe drought stress, increasing the concentration of chitosan reduced the accumulation of phenolic compounds in the roots. The highest state of total tanshinones under no stress, moderate stress, and severe drought stress was observed at 200 mg L^−1^ of chitosan. Chitosan may display diverse effects on adaptation of plants to stress depending on the plant species, application method, concentration, and time of application [35,48]. The hydroxycryptotanshinone and cryptotanshinone contents in both *salvia* species increased with 100 and 200 mg L^−1^ of chitosan foliar application under non-stressed and mildly stressed conditions. Under severe drought stress, the foliar application of 200 mg L^−1^ of chitosan had an increasing effect on the 4CL and DXS2 expressions in *S. abrotanoides.* Further research is warranted to optimize the chitosan application methods, concentrations, and useful times for application under water-limited conditions in the field, as well as to elucidate the molecular mechanisms underneath the observed metabolic plasticity in terms of gene expression and chemical phenotype levels. These findings highlight the complexity of the effects of drought stress and chitosan application on plant traits, necessitating further investigation into the underlying mechanisms.

## Figures and Tables

**Table 1:** Variance analysis of phytochemical traits in *Salvia abrotanoides* and *S yangii* under treatment with water deficit and chitosan.

Mean Squares						
Source of Variation	DF ^1^	Total Tanshinone Content (Root)	Total Phenolic Content (Leaf)	Total Flavonoid Content (Leaf)	Total Phenolic Content (Root)	Total Flavonoid Content (Root)
Species	1	136.85 **	82.47 ^ns^	3.5 *	357.77 **	0.62 ^ns^
Stress	2	141.66 **	51.05 ^ns^	0.95 ^ns^	48.12 **	1.2 *
Chitosan	2	217.3 **	310.26 *	5.86 **	6.18 ^ns^	1.14 *
Species × Stress ^2^	2	4.35 ^ns^	580.83 **	3.58 ^ns^	31.75 *	2.5 **
Species × Chitosan ^3^	2	91.75 **	168.86 ^ns^	0.69 ^ns^	24.20 *	0.069 ^ns^
Stress × Chitosan ^4^	4	118.44 **	615.3 **	3.88 **	43.41 **	1.51 **
Species × Stress × Chitosan ^5^	4	38.56 ^ns^	241.03 *	3.57 **	36.50 **	0.311 ^ns^
Error	36	16.84	81.97	0.502	7.04	0.348

^1^ Df: degrees of freedom; ^2^ Species and stress interaction (two–way interaction); ^3^ Species and chitosan interaction (two–way interaction); ^4^ Stress and chitosan interaction (two–way interaction); ^5^ Species and stress and chitosan interaction (three–way interaction); ns: Not significant; *: Significant at *p* ≤ 0.05; **: Significant at *p* ≤ 0.01.

**Table 2:** (**A**): Mean comparisons of the influence of stress and chitosan (interaction) on phytochemical traits of *Salvia abrotanoides* and *S. yangii*. (**B**): Mean comparisons of species and stress (interaction) on phytochemical traits of *Salvia abrotanoides* and *S. yangii*. (**C**): Mean comparisons of species and chitosan (interaction) on phytochemical traits of *Salvia abrotanoides* and *S. yangi*.

(**A**)					
**Treatments**	**TTC ^1^** **(mg/g DW)**	**TPC ^2^leaf** **(mg TAE/g DW)**	**TFC ^3^leaf** **(mg QE/gDW)**	**TPCroot** **(mg TAE/g DW)**	**TFCroot** **(mg QE/gDW)**
No-stress					
0 mg L^−1^	8.51 ^e^	35 ^e^	2.42 ^d^	10.54 ^bcd^	1.73 ^a^
100 mg L^−1^	18.02 ^b^	57.18 ^a^	5.04 ^a^	10.83 ^bcd^	1.98 ^a^
200 mg L^−1^	23.71 ^a^	43.61 ^cde^	3.34 ^bc^	13.25 ^ac^	2.018 ^a^
Mild stress					
0 mg L^−1^	8.34 ^e^	49.05 ^abc^	4.06 ^b^	12.65 ^abc^	0.6 ^c^
100 mg L^−1^	11.37 ^cde^	45.89 ^bcd^	4.13 ^b^	15.38 ^a^	2.09 ^a^
200 mg L^−1^	13.71 ^bcd^	44.26 ^cde^	3.11 ^cd^	14.45 ^a^	1.77 ^ab^
Severe stress					
0 mg L^−1^	15.47 ^bc^	36.85 ^de^	2.84 ^cd^	14.99 ^a^	1.69 ^ab^
100 mg L^−1^	10.13 ^de^	37.01 ^de^	3.43 ^bc^	8.65 ^d^	1.45 ^ab^
200 mg L^−1^	15.44 ^bc^	56.36 ^ab^	3.66 ^bc^	9.83 ^cd^	1.18 ^bc^
(**B**)					
**Species**	**Stress**	**TPCleaf** **(mg TAE/g DW)**		**TPCroot** **(mg TAE/g DW)**	**TFCroot** **(mg QE/gDW)**
*S. abrotanoides*	N0-stress	37.48 ^c^		15.62 ^a^	2.28 ^a^
	Mild stress	48.10 ^ab^		15.73 ^a^	1.17 ^c^
	Severe stress	45.78 ^abc^		13.22 ^ab^	1.71 ^bc^
*S. yangii*	N0-stress	53.05 ^a^		7.46 ^c^	1.54 ^bc^
	Mild stress	44.70 ^abc^		12.59 ^b^	1.81 ^ab^
	Severe stress	41.03 ^bc^		9.08 ^c^	1.17 ^c^
(**C**)					
**Species**	**Chitosan**	**Total Tanshinone Content (mg/g DW)**		**Total Phenolic Content_Root_** **(mg TAE/g DW)**	
*S. abrotanoides*	0 mg L^−1^	11.62 ^bcd^		16.54 ^a^	
	100 mg L^−1^	11.16 ^cd^		13.14 ^b^	
	200 mg L^−1^	14.01 ^bc^		14.89 ^ab^	
*S. yangii*	0 mg L^−1^	9.93 ^d^		8.91 ^c^	
	100 mg L^−1^	15.18 ^b^		10.09 ^c^	
	200 mg L^−1^	21.23 ^a^		10.13 ^c^	

In each column, the means followed by the same letter are not significantly different according to the LSD test at 0.05. ^1^ Total tanshinone content (TTC); ^2^ Total phenolic content (TPC); ^3^ Total flavonoid content (TFC).

**Table 3:** Various compounds in the mg/g DW of *Salvia abrotanoides* and *S. yangii* under drought conditions and chitosan application.

Species	*S. abrotanoides*									*S. yangii*								
Treatments	Non-Stress			Mild Stress			Severe Stress			Non-Stress			Mild Stress			Severe Stress		
Compositions (mg/gDW)	0	100	200	0	100	200	0	100	200	0	100	200	0	100	200	0	100	200
Tanshinone-IIA	0.448	0.480	1.123	0.368	0.807	0.832	1.040	0.401	0.277	0.496	0.700	0.977	0.455	0.509	1.022	1.757	0.553	0.975
Hydroxy-Cryptotanshinone	4.020	4.320	7.788	5.169	2.736	7.691	8.736	2.587	4.273	2.088	5.533	12.328	4.052	3.643	6.793	4.692	4.139	9.133
Cryptotanshinone	0.667	0.739	0.929	0.533	0.900	0.999	1.277	0.622	0.632	0.305	1.237	1.771	0.684	0.796	0.975	0.910	0.721	1.313
Apigenin (Leaf)	0.067	0.125	0.085	0.092	0.075	0.082	0.110	0.085	0.099	0.085	0.089	0.078	0.067	0.087	0.082	0.682	0.087	0.103
Rosmarinic acid (Leaf)	0.145	11.435	6.674	6.908	4.967	1.737	0.161	4.612	5.065	0.545	9.671	1.871	0.967	8.522	4.309	1.500	0.038	2.281
Gallic acid (Leaf)	0.455	0.059	0.037	0.055	0.062	0.046	0.006	0.458	0.058	0.059	0.066	0.083	0.052	0.049	0.066	0.012	0.255	0.034
Caffeic acid (Leaf)	0.080	0.227	0.188	0.208	0.252	0.098	0.110	0.103	0.246	0.105	0.155	0.192	0.121	0.210	0.218	0.074	0.082	0.318
Chlorogenic acid (Leaf)	0.020	0.328	0.149	0.169	0.201	0.103	0.071	0.034	0.136	0.089	0.060	0.112	0.084	0.099	0.115	0.071	0.169	0.166
Apigenin (Root)	0.068	0.070	0.065	0.057	0.048	0.065	0.060	0.064	0.062	0.057	0.075	0.068	0.076	0.082	0.068	0.056	0.085	0.055
Rosmarinic acid (Root)	3.812	3.051	5.787	4.259	3.863	1.154	1.747	2.254	0.536	1.952	1.956	1.402	1.467	4.004	3.384	2.108	1.348	1.075
Gallic acid (Root)	0.128	0.124	0.106	0.054	0.017	0.020	0.094	0.035	0.148	0.011	0.020	0.039	0.072	0.143	0.018	0.510	0.016	0.053
Caffeic acid (Root)	0.114	0.165	0.191	0.076	0.136	0.144	0.100	0.131	0.084	0.112	0.164	0.159	0.102	0.092	0.420	0.067	0.354	0.086
Chlorogenic acid (Root)	0.039	0.194	0.123	0.061	0.061	0.123	0.026	0.072	0.061	0.069	0.143	0.105	0.067	0.057	0.188	0.077	0.126	0.046

**Table 4:** Correlation coefficients of bioactive components and gene expression under stress conditions and chitosan application.

	Traits	1	2	3	4	5	6	7	8	9	10
1	Tanshinone-IIA	1									
2	Hydroxy-cryptotanshinone	0.48 *	1								
3	Cryptotanshinone	0.54 *	0.86 **	1							
4	KSL ^1^	−0.4	−0.3	−0.4	1						
5	4CL ^2^	0.11	−0.04	0.01	0.14	1					
6	HMGR ^3^	−0.13	0.02	0.12	0.12	0.2	1				
7	DXS ^4^	−0.17	−0.18	−0.15	0.07	0.62 **	0.21	1			
8	TAT ^5^	0.21	0.05	0.16	−0.02	0.62 **	0.005	0.03	1		
9	Total phenolic_leaf_	−0.04	0.05	0.13	0.02	−0.23	0.04	−0.13	−0.08	1	
10	Total phenolic_root_	0.25	0.07	0.12	−0.24	−0.11	−0.14	−0.11	0.16	−0.18	1

* Significant at *p* ≤ 0.05; ** Significant at *p* ≤ 0.01; ^1^ Kaurene synthase-like (KSL); ^2^ 4-Coumarate-CoA ligase gene (4CL); ^3^ 3-Hydroxy-3-methylglutaryl CoA reductase (HMGR); ^4^ 1-Deoxy-D-xylulose 5-phosphate synthase (DXS); and ^5^ Tyrosine aminotransferase gene (TAT).

**Table 5:** Primer sequences of the genes used for real-time PCR.

Target Genes	Primers F/R (5′→3′)
KSL	AATTAGATACATGGAGCGAT
HMGR	ATTTTATACCGATGAACTGC
DXS2	CTTGCCATCATTAATAGCC
TAT	TGCAATATTTATCGCCACT
4Cl	GTTCCCCGACCGTTGCTTC
Elongation Factor	GCACCACCTGATCGTACCC

## Data Availability

The data will be available based on request.
